# Treatment Pattern of Type 2 Diabetes Differs in Two German Regions and with Patients' Socioeconomic Position

**DOI:** 10.1371/journal.pone.0099773

**Published:** 2014-06-10

**Authors:** Teresa Tamayo, Heiner Claessen, Ina-Maria Rückert, Werner Maier, Michaela Schunk, Christine Meisinger, Andreas Mielck, Rolf Holle, Barbara Thorand, Maria Narres, Susanne Moebus, Amir-Abbas Mahabadi, Noreen Pundt, Bastian Krone, Uta Slomiany, Raimund Erbel, Karl-Heinz Jöckel, Wolfgang Rathmann, Andrea Icks

**Affiliations:** 1 Institute of Biometrics and Epidemiology, German Diabetes Center, Leibniz Center for Diabetes Research at Heinrich-Heine-University, Düsseldorf, Germany; 2 Institute of Epidemiology II, Helmholtz Zentrum München, German Research Center for Environmental Health, Neuherberg, Germany; 3 Institute of Health Economics and Health Care Management, Helmholtz Zentrum München, Neuherberg, Germany; 4 German Center for Diabetes Research (DZD e.V.), Neuherberg, Germany; 5 Institute for Medical Informatics, Biometry, and Epidemiology, University Hospital of Essen, University Duisburg-Essen, Essen, Germany; 6 Department of Cardiology, West-German Heart Center, University of Duisburg-Essen, Essen, Germany; 7 Department of Public Health, Faculty of Medicine, Heinrich-Heine University Düsseldorf, Düsseldorf, Germany; Bielefeld Evangelical Hospital, Germany

## Abstract

**Background:**

Diabetes treatment may differ by region and patients' socioeconomic position. This may be particularly true for newer drugs. However, data are highly limited.

**Methods:**

We examined pooled individual data of two population-based German studies, KORA F4 (Cooperative Health Research in the Region of Augsburg, south), and the HNR (Heinz Nixdorf Recall study, west) both carried out 2006 to 2008. To ascertain the association between region and educational level with anti-hyperglycemic medication we fitted poisson regression models with robust error variance for any and newer anti-hyperglycemic medication, adjusting for age, sex, diabetes duration, BMI, cardiovascular disease, lifestyle, and insurance status.

**Results:**

The examined sample comprised 662 participants with self-reported type 2 diabetes (KORA F4: 83 women, 111 men; HNR: 183 women, 285 men). The probability to receive any anti-hyperglycemic drug as well as to be treated with newer anti-hyperglycemic drugs such as insulin analogues, thiazolidinediones, or glinides was significantly increased in southern compared to western Germany (prevalence ratio (PR); 95% CI: 1.12; 1.02–1.22, 1.52;1.10–2.11 respectively). Individuals with lower educational level tended to receive anti-hyperglycemic drugs more likely than their better educated counterparts (PR; 95% CI univariable: 1.10; 0.99–1.22; fully adjusted: 1.10; 0.98–1.23). In contrast, lower education was associated with a lower estimated probability to receive newer drugs among those with any anti-hyperglycemic drugs (PR low vs. high education: 0.66; 0.48–0.91; fully adjusted: 0.68; 0.47–0.996).

**Conclusions:**

We found regional and individual social disparities in overall and newer anti-hyperglycemic medication which were not explained by other confounders. Further research is needed.

## Introduction

Regional differences in treatment patterns, in particular for drug prescriptions, receive growing attention in several countries [1], [2]. However, data are still scarce. This is also true for regional differences in anti-hyperglycemic treatment.

During the last years, new treatment options for type 2 diabetes arose. While newer medications such as glitazones, glinides and insulin analogues enrich treatment options, metformin (biguanides) remains the oral drug of first choice in type 2 diabetes treatment [3], [4]. Little is known about regional differences in prescriptions of newer anti-hyperglycemic drugs.

Furthermore, the association between individuals' socioeconomic position and patterns of medication are of increasing interest. Despite the wide literature on the general topic, socioeconomic factors are rarely examined in association with drug treatment, particularly in the context of anti-hyperglycemic medication. We found only one Canadian study which could show that patients' income had an important impact on the probability of receiving newer thiazolidinediones (TZDs) [5], [6].

In Germany, about 90% of all individuals are covered by a statutory health insurance which reimburses all medical services covering both newer and older diabetes medications [7], [8]. However, in order to provide economic efficiency, benchmarks for budgeting are defined in collective agreements between statutory health insurances and physicians [8]. Resident physicians can also conclude selective contracts with providers of statutory health insurances which may include further tools for guidance. About 10% of the population are privately health insured (e.g. self-employed individuals, civil servants and their family members). These private health insurances impose less economic regulations on physicians and offer some extra services basically to provide more convenience to patients (e.g. single-bed rooms for inpatient treatment, medical attention by a chief physician). For statutory health insured patients, a disease management program (DMP) for diabetes has been implemented in 2002 [9], covering a large proportion of voluntarily participating patients with diabetes. This DMP harmonizes diabetes management and provides financial compensation for (also voluntarily) participating physicians. Within the DMP program quality standards have been defined such as HbA1c targets, prevention of hypoglycemic episodes and other emergency situations, treatment of hypertension, reduction of tobacco consumption among patients, increasing numbers of patients who receive disease-specific education [10]. Physicians are regularly informed about the average achievement of these goals among their patients in comparison to all registered patients. Regarding anti-hyperglycemic treatment, metformin is explicitly recommended in overweight patients with oral monotherapy. However, individual treatment decisions (in order to reach the aforementioned goals) are supported [10]. Thus, it may be assumed that under these conditions, a rather homogenous treatment pattern exists. However, in an earlier study of the Diabetes Collaborative Research of Epidemiologic Studies (DIAB-CORE) consortium, based on pooled individual population-based data, self-reported anti-hyperglycemic medication differed across regions, without showing a clear geographical pattern [11]. Also, the regional population-based studies used for analysis were conducted between 1999 and 2006, when disease management programs were not widespread. In addition, newer treatment options only just arose so that disparities in insulin analogues or newer oral anti-hyperglycemic drugs have not yet been examined.

The aim of our study was to examine (i) if the previously found regional differences in anti-hyperglycemic treatment still exist at a more recent date, (ii) if general regional disparities in treatment patterns exist, e.g. the proportion of patients who receive anti-hyperglycemic drugs, but also drug patterns, e.g. prescription of newer drugs, and (iii) if treatment patterns differ with patients' individual socioeconomic status. We used population-based follow-up data from two German regions, one in the south and one in the west which have been carried out in a comparable time frame between 2006 and 2008.

## Materials and Methods

### Ethics Statement

The Heinz Nixdorf Recall (HNR) study, including the study protocols for participant recruitment, and the informed consent for participants, were approved by the institutional local ethical committees (baseline: Medical faculty University of Essen; follow-up: Medical faculty University of Duisburg-Essen). A quality management system according to European industrial norms (DIN EN ISO 9001:2000) was applied. All participants gave their written consent.

In the KORA studies the participants provided written informed consent. The ethics committee (Bayerische Landesärztekammer) approved the study and approved the consent procedure including patient information materials and consent form.

### Study population and ascertainment of diabetes

Two studies were included: the first follow-up of the Heinz Nixdorf Recall Study (HNR) which was conducted in the adjacent cities of Essen, Bochum and Mülheim of the Ruhr-Area (North Rhine-Westphalia, western Germany) and the first follow-up of the Cooperative Health Research in the Region of Augsburg Survey (KORA F4) study, covering the city of Augsburg and two surrounding rural districts (Bavaria, southern Germany).

4,261 participants attended baseline examinations in KORA S4 (1999–2001; 25–74 years; response 66.8%) [12] and 4.814 participants in HNR (2000–2003; 45–74 years; response 55.8%) [13]. Of these, 3080 participated in the F4 follow-up study in KORA (2006–2008, response 79.6%) [14], and 4,146 in HNR (2005–2008, 86.1% response) [15], [16]. To allow for comparability, people aged at least 50 years at follow-up were included. Further details of the KORA F4 study and HNR have been described elsewhere [16]–[18].

Prevalent diabetes was defined based on self-report of a physician's diagnosis or self-reported anti-hyperglycemic treatment (insulin or oral glucose lowering agents). Study objectives address mainly type 2 diabetes. Since distinction of type 1 and type 2 diabetes is not highly valid in self-reports, subjects with self-reported age at diagnosis before the age of 30 years – possibly cases with type 1 diabetes - were excluded. Self-reported age at diagnosis was ascertained in both studies. Diabetes duration was calculated as the timeframe between age at follow-up and age at diagnosis.

Overall, 199 participants had self-reported diabetes in KORA F4 and 492 in HNR. After exclusion of 26 participants with missing information in relevant data or limited comparability (n = 5 in KORA F4, n = 24 in HNR), 194 individuals with type 2 diabetes from KORA F4 and 468 participants from HNR remained for analysis.

### Assessment and classification of prescribed anti-hyperglycemic drugs

Participants were asked to bring the original packages of all medications used during the seven days prior to the interview to the examination center. Using a scanning system, unique pharmaceutical identifiers were recorded assigning ATC codes (Anatomical Therapeutic Chemical Classification System) which are displayed by 7 characters. The first three characters of the ATC code “A10” indicate any kind of diabetes medication. Under “A10A” all types of insulin are subsumed, while oral anti-hyperglycemic agents start with the code “A10B”.

Following Waugh et al. [3], a drug was considered as “newer” if it belonged to the following groups of glucose lowering drugs:

1. Insulin analogues: Lispro (A10AB04, A10AC04), combinations with Lispro (A10AD04), Aspart (A10AB05, A10AC05, A10AD05), Glulisine (A10AB06), Glargine (A10AE04), Detemir (A10AE05).
2. Newer oral anti-hyperglycemic medications: thiazolidinediones (A10BG: e.g. Rosiglitazone, Pioglitazone), glinides (A10BX: e.g. Repaglinide, Nateglinide), DPP4-inhibitors (A10BH), and combinations of thiazolidinediones or glinides with metformin or glimepiride (A10BD03-A10BD08).

All other anti-hyperglycemic drugs were classified as older medications.

The examined newer anti-hyperglycemic drugs have been introduced shortly before or after year 2000. Since then, both older and newer drugs were available for anti-hyperglycemic treatment in Germany. DPP4-Inhibitors were introduced in 2007 and were not yet used by patients from both cohorts (time of examination 2005/2006–2008). Furthermore, pioglitazone was under restriction only after 2011, so that this drug was still reimbursed by statutory health insurances at the time of both surveys.

### Socioeconomic measures

In our main analysis, we used educational level as indicator of socioeconomic position, as many analyses [19]–[21]. Educational level was assessed by highest self-reported schooling degree achieved at baseline examination. A dichotomous variable was created to indicate high and low educational level. Low educational level was assumed if only junior high school was attended or if no schooling degree has been achieved. High educational level was defined by completed high (higher educational entrance qualification, advanced technical college entrance) or middle educational graduation (general certificate of secondary education or polytechnic grammar school, POS). In other words, completed 10 years of schooling or more were classified as high educational level.

In the former German Democratic Republic (GDR), a POS degree before 1965 was obtained after 8 years of schooling. In 1965 the schooling system was changed and 10 years were needed to achieve a POS degree. Three participants with a POS degree (in HNR) born before 1951 were excluded from the analysis, because of the limited comparability.

Information on monthly net household income as well as on household size was obtained from personal interviews. Following the example of earlier studies within the DIAB-CORE consortium, we calculated the equivalent income according to the Luxembourg Income Study (income/household size) [22], [23].

### Anthropometry, blood pressure, HbA1c, history of stroke, myocardial infarction, and cardiovascular medication

Body mass index was calculated from measured height and weight. In both studies, systolic and diastolic blood pressure was measured by trained personal using a validated automatic device (OMRON HEM 705-CP, OMRON Corporation, Hoofdorp, The Netherlands). Three independent blood pressure measurements were taken with a 3-minute pause in a sitting position on the right arm. The mean of second and third measurement was used for the current analyses.

In HNR, HbA1c was measured by latex agglutination inhibition in EDTA whole blood using the ADVIA Chemistry System. In KORA F4, EDTA plasma was analyzed by high performance liquid chromatography using Menarini HA-8160. Due to these different assessment methods HbA1c values were not considered as statistically comparable between both studies. Nonetheless, we included HbA1c measurements in the regression models especially to determine its confounding effect in stratified analyses.

History of stroke and myocardial infarction was assessed by participant's self-reports (“Did you ever have a myocardial infarction/stroke diagnosed by a physician?”). Cardiovascular treatment was taken from self-reported medication as described above. ATC code “C” indicated any cardiovascular treatment.

### Insurance status, family status and lifestyle measures

Insurance status was assessed in both studies according to patients' self-reports. A binary variable was created separating persons who were privately health insured from those who were statutory health insured. The family situation and marital status were assessed and a dichotomous variable was created for living with a partner (yes/no).

Smoking habits were assessed in an interview situation in both studies. A dichotomous variable was created separating current from ex- and never-smokers. Current smokers needed to smoke at least one cigarette per day. Ex-smokers had smoked at least one cigarette per day in the past, but had quit smoking at least one year ago. Never-smokers had never smoked or had smoked only occasionally (<1 cigarette / day).

In KORA F4, the physical activity level was estimated based on self-reported time per week spent on sports activities during leisure time in summer and winter. Participants were considered physically active if they participated in sports for at least one hour per week and as inactive if they were active for <1 hour per week in summer or winter. In HNR, individuals were also considered as active, if they were active for at least one hour per week but without differentiation between summer and winter activity.

Thresholds for high alcohol consumption were defined for men and women (>20 g/day in women and >40 g/day in men). The calculation of daily alcohol amount was based on weekly consumption of beer, wine and liquor according to Kraus and Augustin [24].

### Statistical analysis

For descriptive analyses, means and standard deviations of all continuous variables as well as proportions and numbers of all categorical covariates were computed in the total population as well as stratified by study and educational level. Likewise, proportions of treatment with anti-hyperglycemic pharmaceutical components were calculated.

We performed two evaluations of the association between educational level and regional disparities with anti-hyperglycemic medication: First, the association with any medication was examined and prevalence ratios (PR) were estimated following Zhou et al. by multivariable poisson regression models with a robust error variance using log link function [25]. Second, this association was examined with newer medication as outcome among participants with any anti-hyperglycemic medication. This methodological approach was chosen due to a high prevalence of both outcomes, whereby an odds ratio calculated from logistic regression models would considerably overestimate the true effect [26]. Univariable models for study, educational level (as main predictors) and all potential confounders were fitted respectively. Additionally, three models were fitted adjusting for (1) age at examination (one year difference in the age groups compared), sex (male vs. female), diabetes duration (years); (2) variables of model 1 plus BMI (kg/m^2^), diastolic and systolic blood pressure (mmHG), HbA1C (% and mmol/mol), stroke (yes vs. no) and myocardial infarction (yes vs. no) in the past; (3) variables of model 2 plus lifestyle factors, i.e. living with a partner (yes vs. no), sports activities (yes vs. no), current smoking (yes vs. no), high alcohol consumption (yes vs. no) and private health insurance (vs. statutory health insurance). In sensitivity analyses, all analyses were repeated with income as measure for socioeconomic position. All models were performed in the total population with type 2 diabetes as well as stratified by region.

Analyses were performed using SAS statistical software version 9.3 (SAS Institute Inc., Cary, NC, USA).

## Results

### Description of the study population

**Table 1** shows the characteristics and patterns of anti-hyperglycemic treatment of participants with type 2 diabetes stratified by region and education. In comparison to KORA F4, participants from HNR more frequently had a higher education, were slightly younger at examination and at diagnosis of diabetes, had a shorter duration of diabetes, had higher systolic and diastolic blood pressure measurements along with a slightly lower BMI. Male preponderance was particularly seen among participants with high education in both studies. Living with a partner and smoking were more common in HNR while high alcohol consumption, sports activities and being privately health insured were more frequent in KORA F4 especially in participants with high education. Regarding diabetes related complications, stroke and cardiovascular treatment were more common in HNR while percentages for myocardial infarction were similar in both studies. Individuals with low education more often had these complications in both studies. For HbA1c (KORA F4: plasma; HNR: whole blood), similar values were found in all strata.

**Table 1:** Characteristics and patterns of anti-hyperglycemic treatment of participants with self-reported type 2 diabetes in the KORA F4 study and the HNR study*.

	Total	KORA	HNR	KORA		HNR		p-value^f)^
				Low education	High education	Low education	High education	
	% (n) or mean (SD)	% (n) or mean (SD)	% (n) or mean (SD)	% (n) or mean (SD)	% (n) or mean (SD)	% (n) or mean (SD)	% (n) or mean (SD)	
Total N	662	194	468	145	49	329	139	
**Description of the study population**								
Low education	71.6 (474)	74.7 (145)	70.3 (329)	-----	-----	-----	-----	
Male sex	59.8 (396)	57.2 (111)	60.9 (285)	**52.4 (76)^b^**	**71.4 (35)^b^**	**56.5 (186)^c^**	**71.2 (99)^c^**	**0.0016**
Age at examination (years)	67.2 (7.3)	**68.2 (7.7)^a^**	**66.8 (7.1)^a^**	68.6 (7.6)	67.1 (8.0)	**67.5 (7.0)^c^**	**65.0 (7.0)^c^**	**0.0003**
Age at diagnosis of diabetes (years)	58.7 (9.8)	59.2 (9.7)	58.6 (9.9)	59.1 (10.1)	59.6 (8.5)	**59.2 (9.7)^c^**	**57.0 (10.2)^c^**	0.1393
Diabetes duration (years)	8.5 (8.0)	9.0 (7.9)	8.2 (8.1)	**9.6 (8.3)^d^**	7.5 (6.3)	**8.3 (8.2)^d^**	8.0 (7.8)	0.1725
BMI (kg/m^2^)	31.0 (5.5)	31.4 (5.5)	30.8 (5.5)	31.6 (5.5)	30.9 (5.6)	31.0 (5.6)	30.1 (5.3)	0.0900
Systolic blood pressure (mmHg)	137.1 (20.8)	**132.8 (20.2)^a^**	**138.8 (20.8)^a^**	**134.7 (21.2)^b,d^**	**127.1 (15.7)^b,e^**	**139.3 (20.7)^d^**	**137.8 (21.3)e**	**<0.0001**
Diastolic blood pressure (mmHg)	76.6 (10.6)	**74.1 (10.2)^a^**	**77.6 (10.7)^a^**	**74.4 (10.8)^d^**	**73.5 (8.5)^e^**	**77.4 (10.7)^d^**	**78.2 (10.6)^e^**	**0.0007**
HbA1c (mmol/mol)**		50.9 (10.8)	51.0 (12.5)	51.4 (10.9)	49.6 (10.6)	51.2 (12.7)	50.5 (12.0)	**
Living with partner	79.6 (527)	75.8 (147)	81.2 (380)	74.5 (108)	79.6 (39)	79.0 (260)	86.3 (120)	0.0907
Current smoker	16.0 (106)	11.9 (23)	17.7 (83)	12.4 (18)	10.2 (5)	18.2 (60)	16.6 (23)	0.2985
High alcohol consumption	7.9 (52)	**11.9 (23)^a^**	**6.2 (29)^a^**	**11.0 (16)^d^**	14.3 (7)	**4.9 (16)^d^**	9.4 (13)	**0.0176**
Sports activity <1 h per week	58.8 (389)	**52.1 (101)^a^**	**61.5 (288)^a^**	**53.1 (77)^d^**	49.0 (24)	**65.7 (216)^c,d^**	**51.8 (72)^c^**	**0.0042**
Previous Stroke	6.8 (45)	4.6 (9)	7.7 (36)	4.8 (7)	4.1 (2)	8.2 (27)	6.5 (9)	0.5470
Previous Myocardial infarction	10.1 (67)	10.3 (20)	10.0 (47)	12.4 (18)	4.1 (2)	11.3 (37)	7.2 (10)	0.2198
Any cardiovascular treatment	83.7 (554)	82.5 (160)	84.2 (394)	83.5 (121)	79.6 (39)	**86.6 (285)^c^**	**78.4 (109)^c^**	0.1269
Privately health insured**	6.2 (39)	**8.3 (16)^a^**	**5.3 (23)^a^**	**6.2 (9)^d^**	14.3 (7)	**1.9 (6)^c,d^**	**13.9 (17)^c^**	**<0.0001**
**Any anti-hyperclycaemic treatment (according to ATC-Codes) incl. multiple treatment in % (n = 499)**								
Any anti-hyperglycemic treatment (insulin, oral or combinations)	75.4 (499)***	**82.5 (160)^a^**	**72.4 (339)^a^**	**85.5 (124)^d^**	73.5 (36)	**73.8 (243)^d^**	69.1 (96)	**0.0065**
All oral anti-hyperglycemic treatment	64.2 (425)	**71.7 (139)^a^**	**61.1 (286)^a^**	71.7 (104)	71.4 (35)	62.3 (205)	58.3 (81)	0.0622
- Metformin	47.7 (316)	49.5 (96)	47.0 (220)	50.3 (73)	46.9 (23)	48.6 (160)	43.2 (60)	0.6431
- Sulfonylureates	25.4 (168)	29.9 (58)	23.5 (110)	31.0 (45)	26.5 (13)	25.8 (85)	18.0 (25)	0.0819
- Metformin+Glinides/Glitazones	2.3 (15)	3.6 (7)	1.7 (8)	3.5 (5)	4.1 (2)	1.8 (6)	1.4 (2)	0.4220
- Acarbose/Miglitol (α-Glucosidaseinhib.)	0.9 (6)	1.6 (3)	0.6 (3)	1.4 (2)	2.0 (1)	**0.3 (1)^c^**	**1.4 (2)^c^**	0.2095
- Glitazone (Thiazolidinediones)	3.3 (22)	5.7 (11)	2.4 (11)	4.1 (6)	10.2 (5)	1.2 (4)	5.0 (7)	**0.0028**
- DPP4-inhibitors	0.5 (3)	0.5 (1)	0.4 (2)	0.7 (1)	0.0 (0)	0.6 (2)	0.0 (0)	1.000
- Glinide	4.1 (27)	3.1 (6)	4.5 (21)	3.5 (5)	2.0 (1)	3.7 (12)	6.5 (9)	0.4959
Treatment with any insulin	20.1 (133)	23.7 (46)	18.6 (87)	25.5 (37)	18.4 (9)	18.8 (62)	18.0 (25)	0.3486
Treatment unknown	0.6 (4)	0.0 (0)	0.9 (4)	0.0 (0)	0.0 (0)	0.9 (3)	0.7 (1)	0.7553
**Newer anti-hyperglycemic treatment among participants with any anti-hyperglycemic medication in % (n = 499)** ***								
All newer drugs (insulin analogue + oral combinations)	23.9 (119)	**30.0 (48)^ a^**	**20.9 (71)^ a^**	**28.2 (35)^d^**	36.1 (13)	**17.3 (42)^c,d^**	**30.2 (29)^c^**	**0.0056**
Any (newer) insulin analogues	12.8 (64)	**18.1 (29)^ a^**	**10.3 (35)^ a^**	**17.7 (22)^d^**	19.4 (7)	**8.6 (21)^d^**	14.6 (14)	**0.0331**
Any newer oral drug	12.8 (64)	15.0 (24)	11.8 (40)	12.9 (16)	22.2 (8)	9.9 (24)	16.7 (16)	0.1016

* Results are percentages (numbers). Significant differences (p<0.05) are highlighted in bold and marked with a superscript character as follows: a) differences between region; b) differences in KORA between both groups of high/low education; c) differences in HNR between both groups of high/low education; d) differences between region in groups of low education; e) differences between region in groups of high education; f) separate column: p-values derived from Kruskal-Wallis test, testing for differences in all four subgroups (south/west, high/low education). Other group comparisons are based on Fisher's exact test for categorical variables and Wilcoxon test for continuous measures.

Abbreviations: KORA =  Cooperative Health Research in the Region of Augsburg study; HNR =  Heinz Nixdorf Recall study; ATC =  Anatomical Therapeutic Chemical Classification System

** Not statistically comparable between the regions due to different blood samples and measurement methods in KORA and HNR and different standard operating procedures;

11 participants missing in HbA1C, 34 missing in insurance status.

*** Percentages based on individuals with any anti-hyperglycemic treatment.

### Treatment groups

About three fourths of all individuals (N = 499) were treated with any anti-hyperglycemic treatment among these 47.7% with metformin, 25.4% with sulfonylureates, and 20.1% with any insulin. In KORA F4, more participants received anti-hyperglycemic treatment than in HNR, in particular individuals with low education. This pattern was similar for oral anti-hyperglycemic treatment and for treatment with any insulin.

Almost one fourth of those receiving any anti-hyperglycemic medication were treated with newer anti-hyperglycemic drugs. This proportion was higher in KORA F4 than in HNR. In contrast to any anti-hyperglycemic treatment, the frequency of newer anti-hyperglycemic treatment was substantially higher among participants with high education. These findings were consistent when considering solely newer insulin analogues as well as newer oral drugs.

Prevalence of insulin as monotherapy did not differ between both studies, while this proportion was substantially higher among individuals with low education in KORA F4 (13.8% vs. 2.0%) (data not shown).

### Determinants of any anti-hyperglycemic treatment in the total population

The results of regression models modeling factors associated with any anti-hyperglycemic medication are shown in **table 2**. In univariable analysis, study location was associated with anti-hyperglycemic medication in such a way that KORA F4 participants had a moderate but significantly higher probability to receive anti-hyperglycemic drugs than participants in HNR (PR:1.14, 95% CI: 1.05–1.24). Adjustment for education, age at examination, sex, diabetes duration, BMI, diastolic and systolic blood pressure, HbA1C, clinical variables (previous myocardial infarction or stroke), lifestyle factors (living with partner, sports activities, smoking, alcohol consumption) and insurance status (PR: 1.12; 1.02–1.22) did not alter the results substantially.

**Table 2:** Factors associated with any anti-hyperglycemic medication (N = 662)*.

Univariable Model	Total	KORA	HNR
	Prevalence Ratio (95% CI)		
Study (KORA vs. HNR)	**1.14(1.05**–**1.24)**	------	------
Low educational level vs. high	1.10(0.99–1.22)	1.16(0.97–1.40)	1.07(0.94–1.22)
Age at examination (years)	1.00(1.00–1.01)	1.01(1.00–1.02)	1.00(0.99–1.01)
Male sex vs. female	1.01(0.93–1.11)	1.04(0.91–1.19)	1.01(0.90–1.13)
Diabetes duration (years)	**1.01(1.01**–**1.02)**	**1.01(1.01**–**1.02)**	**1.01(1.01**–**1.02)**
BMI (kg/m^2^)	**1.01(1.00**–**1.02)**	1.01(1.00–1.02)	1.01(1.00–1.02)
Diastolic blood pressure (mmHg)	1.00(0.99–1.00)	0.99(0.99–1.00)	1.00(0.99–1.00)
Systolic blood pressure (mmHg)	1.00(1.00–1.00)	1.00(0.99–1.00)	1.00(1.00–1.00)
HbA1c (mmol/mol)	**1.01(1.01**–**1.01)**	**1.01(1.00**–**1.01)**	**1.01(1.01**–**1.02)**
Previous stroke (yes vs. no)	0.94(0.78–1.14)	1.08(0.85–1.38)	0.91(0.72–1.16)
Previous MI (yes vs. no)	1.01(0.88–1.17)	1.03(0.85–1.26)	1.00(0.83–1.20)
Living with partner (yes vs. no)	0.95(0.86–1.05)	0.89(0.79–1.02)	1.00(0.86–1.15)
Sports activity <1 h a week (yes vs .no)	1.05(0.96–1.15)	**1.15(1.01**–**1.32)**	1.02(0.91–1.14)
Current smoker (yes vs. no)	0.91(0.80–1.04)	1.00(0.82–1.22)	0.90(0.76–1.06)
High alcohol consumption (yes vs. no)	1.05(0.91–1.22)	1.00(0.82–1.22)	1.05(0.85–1.30)
Private insured (vs. statutory health insured)	1.03(0.86–1.22)	1.07(0.88–1.30)	0.97(0.73–1.27)
**Basic model**			
Low educational level vs. high	1.09(0.98–1.21)	1.14(0.95–1.36)	1.08(0.94–1.23)
Study (KORA vs. HNR)	**1.13(1.03**–**1.22)**	------	------
Age at examination (years)	1.00(0.99–1.00)	1.00(0.99–1.01)	1.00(0.99–1.00)
Male sex vs. female	1.02(0.93–1.11)	1.05(0.93–1.19)	1.00(0.89–1.12)
Diabetes duration (years)	**1.01(1.01**–**1.02)**	**1.01(1.01**–**1.02)**	**1.02(1.01**–**1.02)**
**“Clinical model”: Basic model + BMI + comorbidities + Blood Pressure** **			
Low educational level vs. high	1.08(0.97–1.21)	1.17(0.98–1.39)	1.06(0.93–1.22)
Study (KORA vs. HNR)	**1.13(1.03**–**1.23)**	------	------
Age at examination (years)	1.00(0.99–1.01)	1.00(0.99–1.01)	1.00(0.99–1.01)
Male sex vs. female	1.04(0.95–1.14)	1.11(0.96–1.29)	1.01(0.90–1.13)
Diabetes duration (years)	**1.01(1.01**–**1.02)**	**1.01(1.00**–**1.02)**	**1.01(1.01**–**1.02)**
BMI (kg/m^2^)	1.01(1.00–1.01)	1.01(1.00–1.02)	1.00(0.99–1.01)
Diastolic blood pressure (mmHg)	1.00(0.99–1.00)	1.00(0.99–1.00)	1.00(0.99–1.00)
Systolic blood pressure (mmHg)	1.00(1.00–1.00)	1.00(0.99–1.00)	1.00(1.00–1.01)
HbA1c (mmol/mol)	**1.01(1.01**–**1.01)**	**1.01(1.00**–**1.01)**	**1.01(1.01**–**1.01)**
Previous stroke (yes vs. no)	0.92(0.76–1.12)	1.02(0.81–1.30)	0.90(0.71–1.15)
Previous MI (yes vs. no)	0.98(0.85–1.12)	0.92(0.75–1.13)	1.01(0.84–1.22)
**Full model: “Clinical model”+ Lifestyle + Insurance status** ***			
Low educational level vs. high	1.10(0.98–1.23)	1.17(0.98–1.39)	1.08(0.93–1.26)
Study (KORA vs. HNR)	**1.12(1.02**–**1.22)**	------	------
Age at examination (years)	1.00(0.99–1.01)	1.00(0.99–1.01)	1.00(0.99–1.01)
Male sex vs. female	1.05(0.95–1.17)	1.12(0.95–1.31)	1.02(0.90–1.17)
Diabetes duration (years)	**1.01(1.01**–**1.02)**	**1.01(1.00**–**1.02)**	**1.01(1.00**–**1.02)**
BMI (kg/m^2^)	1.00(1.00–1.01)	1.01(1.00–1.02)	1.00(0.99–1.01)
Diastolic blood pressure (mmHg)	1.00(0.99–1.00)	1.00(0.99–1.01)	1.00(0.99–1.01)
Systolic blood pressure (mmHg)	1.00(1.00–1.00)	1.00(0.99–1.00)	1.00(1.00–1.01)
HbA1c (mmol/mol)	**1.01(1.01**–**1.01)**	**1.01(1.00**–**1.01)**	**1.01(1.01**–**1.02)**
Previous stroke (yes vs. no)	0.90(0.73–1.11)	1.02(0.80–1.30)	0.87(0.67–1.13)
Previous MI (yes vs. no)	1.01(0.89–1.16)	0.93(0.75–1.15)	1.08(0.90–1.29)
Living with partner (yes/no)	0.98(0.88–1.09)	0.92(0.80–1.06)	1.02(0.87–1.20)
Sports activity <1 h a week (yes vs. no)	1.02(0.93–1.11)	1.09(0.97–1.23)	0.98(0.87–1.10)
Current smoker (yes vs. no)	0.90(0.79–1.04)	1.00(0.81–1.24)	0.86(0.72–1.02)
High alcohol consumption(yes vs. no)	1.05(0.90–1.24)	1.03(0.85–1.24)	1.14(0.88–1.47)
Private insured (vs. statutory health insured)	1.07(0.89–1.27)	1.06(0.85–1.32)	1.04(0.78–1.38)

*Results are prevalence ratios (95%CI) calculated from poisson regression models with robust error variance as proposed by Zhou et al.[25]

Abbreviations: KORA =  Cooperative Health Research in the Region of Augsburg study; HNR =  Heinz Nixdorf Recall study; MI =  myocardial infarction.

** 10 missing values (in HbA1c)

*** 37 missing values

Participants with low educational level tended to have a higher albeit not statistically significant probability to receive anti-hyperglycemic medication in univariable analysis (PR: 1.10; 0.99–1.22) as well as in the fully adjusted model (PR 1.10; 0.98–1.23).

In multivariable models, participants with longer diabetes duration had a higher probability to be treated with anti-hyperglycemic drugs (PR and corresponding 95% CI for each year increase in diabetes duration: 1.01; 1.01–1.02). Likewise, the elevation of one unit of HbA1C-value increased this probability (PR(%): 1.12; 1.08–1.16; (mmol/mol): 1.010; 1.007–1.014). Demographic variables such as age and sex as well as all other clinical variables (i.e. blood pressure, previous stroke and myocardial infarction), BMI, lifestyle factors (living with partner, sports activities, current smoker, high alcohol consumption) as well as insurance status had no impact on receiving anti-hyperglycemic treatment.

When stratifying analysis for study region, lower educational level was positively associated with any anti-hyperglycemic medication in both studies in the fully adjusted model, however not reaching level of significance neither in KORA F4 (PR:1.17; 0.98–1.39) nor in HNR (PR: 1.08; 0.93–1.26). In contrast, the associations with diabetes duration and HbA1C remained significant in both study regions. Interaction between education and study region was not significant (p-value for multivariable adjusted interaction term: p = 0.68).

### Determinants of newer anti-hyperglycemic treatment among those with any anti-hyperglycemic treatment

**Table 3** shows the results of the regression models modeling factors associated with newer anti-hyperglycemic medication among the 499 participants with any anti-hyperglycemic medication.

**Table 3:** Factors associated with newer anti-hyperglycemic medication among participants with any anti-hyperglycemic medication (N = 499) (newer vs. older medication)*.

Univariable Model	Total	KORA	HNR
	Prevalence Ratio (95% CI)		
Study (KORA vs. HNR)	**1.43(1.05**–**1.96)**	------	------
Low educational level vs. high	**0.66(0.48**–**0.91)**	0.78(0.47–1.31)	**0.57(0.38**–**0.86)**
Age at examination (years)	1.00(0.98–1.02)	1.00(0.97–1.03)	0.99(0.96–1.02)
Male sex vs. female	0.95(0.69–1.30)	0.72(0.45–1.15)	1.17(0.76–1.81)
Diabetes duration (years)	**1.04(1.03**–**1.06)**	**1.06(1.04**–**1.08)**	**1.03(1.01**–**1.05)**
BMI (kg/m^2^)	1.02(0.99–1.04)	1.03(0.99–1.07)	1.01(0.97–1.04)
Diastolic blood pressure (mmHg)	**0.98(0.97**–**1.00)**	**0.96(0.94**–**0.99)**	1.00(0.98–1.02)
Systolic blood pressure (mmHg)	1.00(0.99–1.00)	0.99(0.98–1.01)	1.00(0.99–1.01)
HbA1c (mmol/mol)	**1.02(1.01**–**1.03)**	1.01(0.99–1.03)	**1.02(1.01**–**1.03)**
Previous stroke (yes vs. no)	0.38(0.13–1.12)	n.e.	0.58(0.20–1.70)
Previous MI (yes vs. no)	0.81(0.45–1.44)	0.37(0.10–1.37)	1.14(0.60–2.17)
Living with partner (yes vs. no)	1.07(0.72–1.58)	1.35(0.74–2.47)	0.95(0.56–1.59)
Sports activity <1 h a week(yes vs. no)	1.02(0.74–1.41)	0.87(0.54–1.39)	1.20(0.77–1.87)
Current smoker (yes vs. no)	0.77(0.46–1.26)	0.32(0.09–1.22)	1.05(0.60–1.82)
High alcohol consumption (yes vs. no)	1.14(0.67–1.94)	0.86(0.39–1.91)	1.33(0.65–2.72)
Private insured (vs. statutory health insured)	**2.00(1.28**–**3.12)**	1.49(0.77–2.87)	**2.40(1.31**–**4.39)**
**Basic model**			
Low educational level vs. high	**0.60(0.44**–**0.83)**	0.62(0.37–1.02)	**0.56(0.37**–**0.87)**
Study (KORA vs. HNR)	**1.50(1.10**–**2.03)**	------	------
Age at examination (years)	0.99(0.97–1.01)	0.98(0.95–1.01)	0.99(0.96–1.02)
Male sex vs. female	0.87(0.65–1.18)	0.66(0.43–1.02)	1.07(0.70–1.63)
Diabetes duration (years)	**1.05(1.03**–**1.06)**	**1.07(1.04**–**1.09)**	**1.04(1.02**–**1.06)**
**“Clinical model”: Basic model + BMI + comorbidities + Blood Pressure** **			
Low educational level vs. high	**0.61(0.44**–**0.85)**	0.77(0.47–1.24)	0.52(0.33–0.80)
Study (KORA vs. HNR)	**1.48(1.08**–**2.02)**	------	------
Age at examination (years)	0.99(0.97–1.02)	0.96(0.93–1.00)	1.01(0.98–1.05)
Male sex vs. female	0.94(0.67–1.31)	0.94(0.57–1.54)	1.05(0.67–1.65)
Diabetes duration (years)	**1.04(1.02**–**1.06)**	**1.06(1.03**–**1.08)**	**1.03(1.01**–**1.05)**
BMI (kg/m^2^)	1.01(0.98–1.04)	1.04(1.00–1.08)	1.00(0.96–1.03)
Diastolic blood pressure (mmHg)	0.99(0.96–1.01)	**0.95(0.91**–**0.98)**	1.01(0.98–1.04)
Systolic blood pressure (mmHg)	1.00(0.99–1.01)	1.01(0.99–1.03)	1.00(0.98–1.01)
HbA1c (mmol/mol)	**1.02(1.01**–**1.03)**	1.01(0.99–1.03)	**1.03(1.01**–**1.04)**
Previous stroke (yes vs. no)	0.30(0.08–1.15)	n.e.	0.47(0.13–1.70)
Previous MI (yes vs. no)	0.78(0.43–1.42)	0.26(0.07–0.98)	1.30(0.66–2.57)
**Full model: “Clinical model”+ Lifestyle + Insurance status** ***			
Low educational level vs. high	**0.68(0.47**–**1.00)**	0.82(0.50–1.37)	**0.57(0.33**–**0.98)**
Study (KORA vs. HNR)	**1.52(1.10**–**2.11)**	------	------
Age at examination (years)	0.99(0.97–1.02)	0.96(0.93–1.00)	1.01(0.98–1.05)
Male sex vs. female	0.86(0.59–1.25)	0.78(0.45–1.35)	1.01(0.59–1.72)
Diabetes duration (years)	**1.03(1.02**–**1.05)**	**1.05(1.03**–**1.08)**	1.02(1.00–1.05)
BMI (kg/m^2^)	1.02(0.99–1.05)	1.04(0.99–1.08)	1.01(0.97–1.05)
Diastolic blood pressure (mmHg)	0.99(0.96–1.01)	**0.94(0.91**–**0.98)**	1.01(0.97–1.04)
Systolic blood pressure (mmHg)	1.00(0.99–1.02)	1.01(0.99–1.03)	1.00(0.98–1.02)
HbA1c (mmol/mol)	**1.02(1.00**–**1.03)**	1.02(1.00–1.04)	**1.02(1.00**–**1.04)**
Previous stroke (yes vs. no)	0.17(0.02–1.17)	n.e.	0.30(0.04–2.03)
Previous MI (yes vs. no)	0.82(0.45–1.50)	**0.25(0.07**–**0.87)**	1.42(0.71–2.85)
Living with partner (yes vs. no)	1.29(0.83–2.00)	1.57(0.84–2.94)	0.97(0.52–1.83)
Sports activity <1 h a week(yes vs. no)	1.03(0.74–1.43)	0.88(0.55–1.39)	1.12(0.69–1.81)
Current smoker (yes vs. no)	0.82(0.48–1.40)	0.38(0.11–1.34)	1.11(0.59–2.09)
High alcohol consumption(yes vs. no)	0.84(0.42–1.67)	0.81(0.35–1.83)	0.82(0.29–2.29)
Privately insured (vs. statutory health insured)	**2.05(1.25**–**3.36)**	1.67(0.80–3.49)	**2.44(1.23**–**4.81)**

*Results are prevalence ratios (95%CI) calculated from poisson regression models with robust error variance as proposed by Zhou et al. [25]. Significant differences (p>0.05) are highlighted in bold.

Abbreviations: KORA =  Cooperative Health Research in the Region of Augsburg study; HNR =  Heinz Nixdorf Recall study; MI =  myocardial infarction, n.e.  =  not estimable.

** 11 missing values (in HbA1c).

*** 45 missing values.

In univariable models, KORA F4 participants had a significantly higher probability to receive newer glucose lowering drugs (PR 1.43; 95% CI: 1.05–1.96). This association remained significant after adjustment for all potential confounders (PR: 1.52; 1.10–2.11). In contrast, persons with low educational level were significantly less frequently treated with newer anti-hyperglycemic drugs compared to those with high education (univariable PR: 0.66; 0.48–0.91), which was also true after multivariable adjustment: (PR: 0.68; 0.47–0.996).

In fully adjusted models diabetes duration (PR: 1.03; 1.02–1.05), HbA1C (PR: 1.02; 95% CI: 1.004–1.03) and being private health insured (PR: 2.05; 95% CI: 1.25–3.36) was also positively associated with newer anti-hyperglycemic medication. Again, age and sex were not associated with newer anti-hyperglycemic treatment as well as BMI, blood pressure, previous myocardial infarction and lifestyle factors.

After stratification for study region, the probability to receive a newer anti-diabetic treatment among participants with lower education was significantly lower only in HNR (KORA F4: 0.82; 95% CI: 0.50–1.37; HNR: 0.57; 0.33–0.98). Regarding covariates, an inconsistent pattern was found. In HNR, HbA1C (PR (%): 1.27; 1.05–1.54; (mmol/mol): 1.023; 1.005–1.041) and being privately health insured were positively associated with newer anti-hyperglycemic treatment (PR: 2.44; 1.23–4.81), while in KORA F4, diabetes duration increased this probability (PR: 1.05; 1.03–1.08). In contrast, negative associations were observed in KORA F4 with diastolic blood pressure (PR for one unit increase: 0.94; 0.91–0.98) as well as previous myocardial infarction (PR: 0.25; 0.07–0.87). The interaction term between education and study region was not significant (p = 0.42).

As further clinical variables, HbA1 and diabetes duration increased the possibility to receive (any or newer) anti-hyperglycemic medication in both studies. Adding both variables to the models did not confound the association of anti-hyperglycemic treatment with region or SES.

### Sensitivity analysis

When we used equivalent income (income/household members) instead of education, the probability of receiving any anti-hyperglycemic medication was not associated with SES in the whole population as well as in stratified analyses (PR for an increase of 100€ income: whole population 1.00; 0.99–1.01; KORA F4 0.99; 0.98–1.005; HNR 1.00; 0.99–1.01). Income was also associated with newer anti-hyperglycemic treatment in KORA F4 (PR for an increase of 100€ income: whole population 1.02; 0.996–1.04; KORA F4 1.06; 1.03–1.09; HNR 1.00; 0.98–1.03). In the fully adjusted models PRs of all other variables did not materially change (data not shown).

## Discussion

### Main findings and implications

In this cross-sectional examination based on pooled individual data from two population-based studies – one in the south (KORA F4) and one in the west (HNR) of Germany – the probability to receive anti-hyperglycemic drugs as well as to receive newer glucose lowering drugs such as insulin analoga, TZDs, or glinides was substantially higher in the south of Germany. Regarding socioeconomic differences, individuals with lower educational level tended to have a higher probability to receive anti-hyperglycemic drugs than their better educated counterparts. However, the association was not significant. Among those with any anti-hyperglycemic medication, individuals with lower educational level had a significantly lower probability to receive newer anti-hyperglycemic drugs than their better educated counterparts. In region-stratified analyses the latter effect was only significant in HNR, however, the overall pattern was similar in both studies. HbA1c and diabetes duration were further independent predictors for anti-hyperglycemic medication in both studies. However, this association could not explain the regional differences in anti-hyperglycemic medication and the difference in high and low SES groups (especially HNR). Furthermore, the regional and socioeconomic differences remained after adjusting for other individual factors available for analysis (such as BMI, lifestyle or complications such as myocardial infarction and stroke).

Importantly, the older anti-hyperglycemic drug metformin remains the oral drug of first choice in current clinical guidelines [27]. These guideline recommendations emphasize the need for individualized treatment decisions which are influenced by clinical decisions as well as other patient characteristics and which in consequence can be responsible for the prescription of newer anti-hyperglycemic drugs. As an example, age, Hba1c levels, expected treatment efforts, diabetes related complications, and co-morbidities of patients merit attention. Disadvantages of metformin such as gastrointestinal side effects, vitamin B12 deficiency, and chronic kidney disease may guide treatment choice towards newer medications [27]. A decision for glitazone includes severe adipositas (insulin resistance) [28]. Furthermore, before 2011, TZDs (pioglitazone) were not recommended for patients with cardiovascular or hepatic disease. However, since 2011, TZDs are under restriction in Germany and are currently not reimbursed by statutory health insurances.

We controlled for some of these variables in our study. However, detailed information on complications other than myocardial infarction and stroke were not available in a highly comparable way for a pooled analysis, so that individual treatment decisions were irreproducible. Nevertheless, the south of Germany is a region with lower overall mortality, lower blood pressure and lower type 2 diabetes prevalence than the west [29], [30]. Thus, given the overall trend for a healthier population in the south, mere clinical decisions are not likely to have caused the regional differences we found.

We could not find any explanations for our findings. In Germany, as well as in most western European countries, almost all individuals are members of a health insurance and have almost free access to the majority of medical services. Exceptions are medications given as over-the-counter medication and for diseases with low severity, e.g. cold, which are paid by the patients. Overall, private expenditures account for about 15% of the health care expenditures [7]. Diabetes treatment in Germany should be rather standardized, in particular since the introduction of disease management programs. However, regional differences with respect to health care services are likely. For example in more rural regions, the availability of specialized diabetes care might be lower compared to urban areas [31]. Respective analyses are planned for the future. In addition, local health care practice including e.g. the screening frequency or generally the awareness of the disease in a population might affect the proportion of undiagnosed diabetes in a region possibly also causing regional differences in sample characteristics.

It could be argued that regional and social discrepancies for newer glucose lowering drug use could be mediated at least in part by health insurance status. Persons with a high income or those who are self-employed are free to take out private health insurance covering extra services of medical care. While statutory health insurances impose a limit on GPs and specialized diabetes practitioners for prescriptions, private health insurance companies are more likely to accept the higher costs for newer medications. Besides cost reasons, individuals with private health insurance might differ from those insured statutorily in such a way that they might participate more actively in treatment decisions and claim for newer medications.

In our study, we found a higher proportion of anti-hyperglycemic drug intake among privately health insured persons compared to statutorily health insured participants, which was significantly increased for newer anti-hyperglycemic medication. Adjusting for health insurance status did not alter the association between education and anti-hyperglycemic medication substantially. These findings could emphasize that persons with a higher education in general might receive newer drugs more frequently irrespective of their status of insurance.

Interestingly, when we used equivalent income as an indicator for socioeconomic status, the probability for receiving any anti-hyperglycemic medication was not associated with SES. However, there was an association with newer anti-hyperglycemic medication which is also more expensive. Similar results were reported in a recent study from Sweden, where drug utilization was associated with education, but not with income [32]. The authors could not explain their findings. They suggest that medication may be influenced particularly by the interaction between physician and patient, and that this interaction may depend on patients' education more than on patients' income level.

### Comparison to other studies

Despite the interest in geographic differences in health care spending and treatment patterns, literature on the contribution of structural deprivation and individual socioeconomic status on anti-hyperglycemic treatment is scarce. Social gradients in treatment with certain medications or diet alone have been found earlier in a Canadian study [33]. Prescription of metformin and sulfonylureas was higher in lower income groups, while “diet-alone” was more often treatment option in higher income quintiles than in lower ones. Another Canadian study based on reimbursement data indicated, that high income groups were more likely to receive restricted medications such as thiazolidinediones (TZDs) compared to low income groups [6], similar to our study. The authors could not explain their finding. Regional disparities in prescription patterns based on insurance data have been described earlier for the prescription prevalence of antibiotic use. A recent German study showed a regional variation of 19–53% of antibiotic use in children which was partly explained by regional deprivation (especially by regional income and occupational deprivation) [34].

Structural differences of health care supply which have recently been reported for Germany might also be relevant for our findings [35]. The authors analyzed if regional health care utilization met the expected needs (equity index  = 1). They could show that factors of health care supply such as physician density and physician contacts explained 49% of health care utilization. A high physician density and a high number of physician contacts was associated with a higher health care utilization beyond the expected needs (equity index below 1). On the other hand, a high number of social welfare recipients in a region was associated with a lower utilization. Regarding our study areas, for Augsburg and its rural surroundings a low equity index was calculated (utilization exceeded the needs) while the equity index was close to 1 in the urban areas of the HNR study. Therefore, structural differences by region such as a higher physician density and a higher number of physician contacts might be important factors contributing to the higher overall anti-hyperglycemic medication use in KORA F4 which should be addressed in further studies.

### Strenghts and limitations

Our study has several limitations. First, we could not examine if direct contracts between general practitioners and health insurance companies, which vary across regions, might have had an impact on treatment decisions. Second, as described above, clinical information on participants was limited. Thus, we could not evaluate if treatment patterns follow clinical guidelines and correspond with indications for newer treatment options. Furthermore, cases with cardiovascular events (myocardial infarction, stroke) were too low in some subgroups so that statistical power was insufficient to detect associations with treatment decisions. Finally, some variables, such as HbA1c, were not exactly comparable between the two studies.

The strengths of our study are highly standardized measurement techniques carried out by trained personnel (e.g. for anthropometry and blood pressure) and the application of very similar, standardized interviews and questionnaires. Sampling frames of both population-based studies aimed for a high representativeness of the data. Furthermore, both studies used a similar scanning system to assign unique pharmaceutical identifiers (ATC codes) to the medication packages brought to the interview date.

In conclusion, we found regional disparities in any and in newer anti-hyperglycemic treatment in Germany. Lower social status was also associated with a lower probability to receive newer anti-hyperglycemic drugs which was especially observed in the Ruhr area (HNR). Overall, these differences were not explained by age, sex, BMI, and lifestyle factors such as sports activities or smoking as well as insurance status. Of note, the disparities in treatment with newer anti-hyperglycemic drugs we found do not implicate regional or social disparities in quality of care. Further research is needed to explain these findings. Especially, studies are warranted that include a larger number of patients and further geographic regions.
